# Prognostic Factors in Metastatic Urothelial Carcinoma Treated Without Methotrexate, Vinblastine, Doxorubicin, and Cisplatin (M-VAC): A Single-Center Retrospective Study

**DOI:** 10.7759/cureus.95066

**Published:** 2025-10-21

**Authors:** Irfan Bugday, Mevlude Inanc, Metin Ozkan, Oktay Bozkurt, Ramazan Cosar, Sedat T Firat, Emel Mutlu, Murat Eser, Ahmet K Disli, Muhammet Cengiz

**Affiliations:** 1 Medical Oncology, Erzurum City Hospital, Erzurum, TUR; 2 Medical Oncology, Erciyes University Faculty of Medicine, Kayseri, TUR

**Keywords:** ecog performance status and prognosis, inflammatory markers in bladder cancer, metastatic bladder cancer, prognostic factors, survival outcomes in urothelial carcinoma

## Abstract

Background

Bladder cancer represents the most prevalent malignancy of the urinary tract. This study aimed to develop a prognostic model for patients with metastatic urothelial carcinoma.

Methodology

This retrospective study included a total of 49 patients who received chemotherapy for metastatic bladder cancer. Clinical and pathological parameters were analyzed to evaluate their prognostic value.

Results

The median progression-free survival (PFS) was calculated as 8.20 months. Patients with an Eastern Cooperative Oncology Group (ECOG) performance status (PS) of 0 had a significantly longer PFS (21.83 months) compared to those with a PS of 1 (5.20 months; p < 0.001). PFS was also significantly longer in patients with transitional epithelial cell carcinoma (10.87 months) compared to those with non-transitional epithelial carcinoma (4.90 months; p = 0.012). Additionally, patients with normal baseline hemoglobin levels demonstrated a longer PFS (12.57 months) than anemic patients (5.20 months; p = 0.002). The median overall survival (OS) was calculated as 15.80 months. Patients with a body mass index (BMI) <25 kg/m² had a median OS of 11.20 months, whereas those with a BMI >25 kg/m² had an OS of 21.30 months (p = 0.003). Patients with transitional epithelial carcinoma had a median OS of 16.30 months, compared to 12.33 months in patients with non-transitional epithelial carcinoma (p = 0.014). Similarly, patients with normal hemoglobin levels had a significantly longer OS (21.30 months) than those with anemia (11.73 months; p = 0.011).

Conclusions

Our findings demonstrate that a baseline ECOG PS of 0 is significantly associated with improved PFS and OS in patients with metastatic urothelial carcinoma. Furthermore, lower pretreatment hemoglobin levels were found to be associated with poorer survival outcomes. The study also identified high white blood cell count, normal platelet count, low neutrophil-to-lymphocyte ratio and platelet-to-lymphocyte ratio, low albumin, and low lactate dehydrogenase levels as favorable prognostic indicators in this patient population. These results contribute to the growing body of literature aimed at optimizing prognostic assessment in metastatic bladder cancer.

## Introduction

Bladder cancer is the most common malignancy of the urinary tract, predominantly affecting individuals over 55 years of age, with an average age at diagnosis of 73 years. The disease is significantly more common in men, who are three to four times more likely to be diagnosed than women [1]. Established risk factors include male sex, older age, Caucasian ethnicity, exposure to carcinogenic chemicals (e.g., arsenic), pelvic radiation history, use of cytotoxic agents such as cyclophosphamide, recurrent bladder infections, family or personal history of bladder cancer, and tobacco use [1].

At initial presentation, approximately 5% of cases are metastatic. Additionally, nearly 50% of patients undergoing cystectomy for localized disease eventually experience recurrence, with distant metastases being more common than local recurrence [2]. Before modern chemotherapy, survival for patients with recurrent or metastatic disease was limited to approximately six months [3].

The M-VAC regimen (methotrexate, vinblastine, doxorubicin, and cisplatin) demonstrated high overall response rates and a median survival of 13 months [4]. A phase III trial comparing M-VAC with gemcitabine-cisplatin (GC) showed similar outcomes (response rates of 49% vs. 46%, median survival of 14.8 vs. 13.8 months), with GC exhibiting a more favorable toxicity profile [5].

A prognostic model based on 203 patients treated with M-VAC identified low Karnofsky performance score (<80) and visceral metastasis as negative prognostic factors [6]. However, validated prognostic tools for patients receiving first-line therapies other than M-VAC are lacking in routine clinical practice.

In this retrospective study, we analyzed clinical and pathological data from patients treated with alternative first-line chemotherapy regimens for metastatic urothelial carcinoma. We aimed to develop a prognostic model by comparing outcomes between patients with and without disease progression.

## Materials and methods

Patients

This retrospective study included 49 patients who received first-line chemotherapy for metastatic bladder cancer between January 1, 2015, and June 30, 2023. Patients without histologically confirmed metastatic urothelial carcinoma or those who received M-VAC regimens were excluded. The study was approved by the Erciyes University Clinical Research Ethics Committee (decision date/number: 23.08.2023/556), and the requirement for informed consent was waived due to the retrospective design.

Data collection

Patient records were reviewed to identify recurrence or metastasis during follow-up. Demographic, clinical, laboratory, and radiological data were extracted. Chemotherapy regimens included cisplatin- or carboplatin-based combinations and gemcitabine-paclitaxel or single-agent paclitaxel, selected according to patient performance status and comorbidities. Progression-free survival (PFS) and overall survival (OS) were calculated using SPSS version 22.0 (IBM Corp., Armonk, NY, USA). Missing data were handled using the state method (e.g., complete-case analysis or imputation).

Statistical analysis

Kaplan-Meier survival curves were generated to estimate PFS and OS, and differences between groups were assessed using the log-rank test. Univariate analysis identified potential prognostic factors, with variables with a p-value <0.05 included in multivariate Cox proportional hazards regression to determine independent predictors. Cut-off values for continuous variables (e.g., white blood cell (WBC) count, platelet-to-lymphocyte ratio (PLR), and neutrophil-to-lymphocyte ratio (NLR)) were determined using receiver operating characteristic (ROC) curve analysis. Proportional hazards assumptions were tested for all Cox models.

## Results

A total of 49 patients were included, with 48 (98%) males and 1 (2%) female. The median age was 68 years (range = 39-86 years). Patient characteristics are summarized in Table 1.

**Table 1:** Demographic and clinical characteristics of the patients. ECOG = Eastern Cooperative Oncology Group; PS = performance status

Variable	Category	n (%)
ECOG PS	0	19 (38.8%)
	1	30 (61.2%)
Pathology	Transitional	28 (57.1%)
	Papillary	17 (34.7%)
	Squamous	4 (8.1%)
Chemotherapy regimen	Cisplatin plus gemcitabine	20 (40.8%)
	Carboplatin plus gemcitabine	18 (36.7%)
	Gemcitabine plus paclitaxel	3 (6.1%)
	Single-agent paclitaxel	8 (16.3%)
Metastasis site	Lymph node	36 (73.5%)
	Lung	26 (53.1%)
	Liver	6 (12.2%)
	Bone	24 (49.0%)
	Intra-abdominal implant	6 (12.2%)
Response to chemotherapy	Complete response	1 (2.0%)
	Partial response	12 (24.5%)
	Stable disease	26 (53.1%)
	Progressive disease	10 (20.4%)

**Figure 1:**
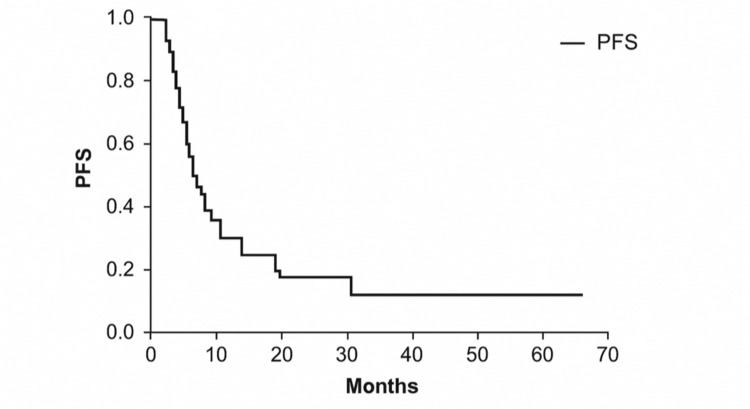
PFS of patients with metastatic urothelial carcinoma. PFS = progression-free survival

The median PFS was 8.20 months (95% confidence interval (CI) = 6.65-9.75) (Figure 1). Patients with Eastern Cooperative Oncology Group (ECOG) performance status (PS) of 0 had longer PFS compared to those with a PS of 1 (21.83 vs. 5.20 months, p < 0.001). Transitional epithelial carcinoma and platinum-based chemotherapy were associated with longer PFS (10.87 vs. 4.90 months, p = 0.012; 10.87 vs. 3.53 months, p < 0.001, respectively). Patients with normal baseline hemoglobin, platelet counts, albumin, and lactate dehydrogenase (LDH) levels also demonstrated improved PFS. Lower NLR (<3.09) and PLR (<196.75) were linked to longer PFS. Treatment-related neutropenia and thrombocytopenia were associated with improved outcomes. Conversely, metastasis to lymph nodes, lung, or bone, and having ≥2 metastatic sites were linked to shorter PFS. Maintenance gemcitabine use prolonged PFS (15.37 vs. 6.47 months, p = 0.012).

**Figure 2:**
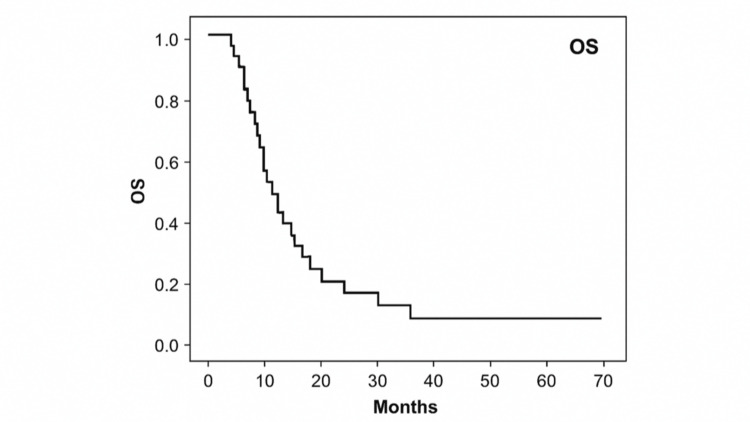
OS of patients with metestatic urothelial carcinoma. OS = overall survival

The median OS was 15.80 months (95% CI = 13.46-18.13) (Figure 2). Longer OS was observed in patients with an ECOG PS of 0 (31.10 vs. 11.73 months, p < 0.001), transitional epithelial carcinoma, body mass index (BMI) >25 kg/m², platinum-based therapy, normal hemoglobin, and normal platelet counts. Lower NLR and PLR and normal albumin and LDH were also favorable. Adverse metastasis sites (lymph node, lung, ≥2 sites) were associated with reduced OS, while maintenance gemcitabine showed a trend toward longer OS.

Multivariate Cox regression identified an ECOG PS of 0, transitional epithelial pathology, anemia, bone metastasis, and having ≥2 metastatic sites as independent prognostic factors for PFS and OS (Table 2).

**Table 2:** Multivariate analysis of PFS and OS. PFS = progression-free survival; OS = overall survival; CI = confidence interval; ECOG = Eastern Cooperative Oncology Group; PS = performance status; WBC = white blood cell; PLR = platelet-to-lymphocyte ratio; NLR = neutrophil-to-lymphocyte ratio

Variable	Multivariate PFS OR (95% CI)	P-value	Multivariate OS OR (95% CI)	P-value
ECOG PS: 0	7.70 (2.80–21.30)	<0.001	9.80 (3.04–31.55)	<0.001
Transitional epithelial pathology	2.23 (1.01–4.91)	0.046	2.99 (1.39–6.39)	0.005
Anemia previous treatment	5.78 (2.31–14.41)	<0.001	2.66 (1.17–6.02)	0.019
Thrombocytopenia previous treatment	2.67 (1.01–7.02)	0.046	1.41 (0.49–4.06)	0.516
Thrombocytopenia with treatment	0.93 (0.39–2.20)	0.876	0.42 (0.17–1.07)	0.071
Neutropenia with treatment	1.38 (0.52–3.65)	0.505	2.14 (0.75–6.09)	0.153
WBC >8.28	1.13 (0.48–2.66)	0.775	1.07 (0.38–2.98)	0.892
PLR <196.75	0.85 (0.28–2.58)	0.779	1.63 (0.47–5.62)	0.433
NLR <3.09	0.79 (0.27–2.33)	0.674	0.39 (0.12–1.25)	0.115
Bone metastasis	2.24 (0.75–6.74)	0.148	3.66 (1.35–9.59)	0.010
Lung metastasis	2.50 (0.84–7.41)	0.097	0.65 (0.24–1.80)	0.417
Number of metastasis sites >2	3.64 (1.14–11.61)	0.029	4.15 (1.26–13.70)	0.019

## Discussion

In patients with advanced or metastatic urothelial carcinoma who are not candidates for emerging therapies such as enfortumab vedotin plus pembrolizumab, cisplatin-based chemotherapy remains an effective first-line option. This study evaluated clinical and laboratory prognostic factors in 49 patients, 85% of whom received platinum-based regimens.

Consistent with prior M-VAC studies, an ECOG PS of 0 was strongly associated with longer PFS and OS, highlighting the importance of functional status assessment before therapy initiation [7]. Baseline anemia was confirmed as a negative prognostic factor, extending prior observations from platinum-refractory settings to platinum-sensitive patients [8].

Patients with transitional epithelial histology exhibited superior survival outcomes compared to other subtypes, suggesting its relevance as a prognostic marker. Higher BMI showed a trend toward better outcomes but did not reach significance in multivariate analysis, likely due to the small, single-center cohort.

Routine laboratory values, including WBC count, platelet count, NLR, PLR, albumin, and LDH, were prognostic in univariate analyses but not in multivariate models, possibly reflecting limited statistical power. As expected, multiple metastatic sites were associated with poorer survival. Interestingly, chemotherapy-induced neutropenia and thrombocytopenia correlated with improved outcomes, possibly indicating a stronger treatment response in some patients. Gemcitabine maintenance after initial platinum therapy also appeared to prolong PFS and OS.

Limitations of this study include its retrospective, single-center design and modest sample size, which may limit generalizability. Despite these constraints, the findings emphasize that readily available clinical and laboratory markers can provide useful prognostic information. Validation in larger, multicenter cohorts and integration with molecular biomarkers are warranted to strengthen prognostic models for routine clinical use.

## Conclusions

Baseline ECOG PS and hemoglobin level remain key prognostic indicators in patients with advanced or metastatic urothelial carcinoma receiving platinum-based chemotherapy. Histologic subtype, metastatic burden, chemotherapy-induced hematologic toxicities, and maintenance therapy may also influence outcomes and warrant further study. Despite the limitations of a single-center, small cohort, readily available clinical and laboratory parameters can help guide prognostication in routine practice. Larger, multicenter studies are needed to validate these findings and refine prognostic models for individualized treatment strategies.

## References

[1] (2025). American Cancer Society. Key statistics for bladder cancer. https://www.cancer.org/cancer/types/bladder-cancer/about/key-statistics.html?utm_source.

[2] (2024). NIH NCI: Surveillance, Epidemiology, and End Results Program. Cancer stat facts: bladder cancer. https://seer.cancer.gov/statfacts/html/urinb.html.

[3] Babaian RJ, Johnson DE, Llamas L, Ayala AG (1980). Metastases from transitional cell carcinoma of urinary bladder. Urology.

[4] Sternberg CN, Yagoda A, Scher HI (1985). Preliminary results of M-VAC (methotrexate, vinblastine, doxorubicin and cisplatin) for transitional cell carcinoma of the urothelium. J Urol.

[5] von der Maase H, Hansen SW, Roberts JT (2000). Gemcitabine and cisplatin versus methotrexate, vinblastine, doxorubicin, and cisplatin in advanced or metastatic bladder cancer: results of a large, randomized, multinational, multicenter, phase III study. J Clin Oncol.

[6] Bajorin DF, Dodd PM, Mazumdar M (1999). Long-term survival in metastatic transitional-cell carcinoma and prognostic factors predicting outcome of therapy. J Clin Oncol.

[7] von der Maase H, Sengelov L, Roberts JT (2005). Long-term survival results of a randomized trial comparing gemcitabine plus cisplatin, with methotrexate, vinblastine, doxorubicin, plus cisplatin in patients with bladder cancer. J Clin Oncol.

[8] Bellmunt J, Choueiri TK, Fougeray R (2010). Prognostic factors in patients with advanced transitional cell carcinoma of the urothelial tract experiencing treatment failure with platinum-containing regimens. J Clin Oncol.

